# Naomaitai Ameliorated Brain Damage in Rats with Vascular Dementia by PI3K/PDK1/AKT Signaling Pathway

**DOI:** 10.1155/2019/2702068

**Published:** 2019-02-05

**Authors:** Kui Huang, Lei Shen, Tieming Niu, Ying Zhao, Jiucun Fu, Yunpeng Cao

**Affiliations:** ^1^Department of Neurology, The First Affiliated Hospital of China Medical University, Shenyang, Liaoning 110001, China; ^2^Department of Geriatric Medicine, Shenyang Red Cross hospital, Shenyang Liaoning 110000, China; ^3^Department of Urologic Department, Shenyang Red Cross hospital, Shenyang Liaoning 110000, China; ^4^Department of Health Management, Shenyang Red Cross hospital, Shenyang Liaoning 110000, China

## Abstract

**Background/Aims:**

Naomaitai can improve blood perfusion and ameliorate the damage in the paraventricular white matter. This study was focused on observing the neuroprotective effect of Naomaitai on the vascular dementia of rat and exploring the action mechanism of PI3K/PDK1/AKT signaling pathway.

**Methods:**

A vascular dementia model of rats was established by permanent, bilateral common carotid artery occlusion. Rats' behavior was tested by Neurological deficit score and the Morris water maze. The pathology and apoptosis were detected through HE staining and TUNEL assay. Myelin sheath loss and nerve fiber damage were detected by LFB staining. Inflammatory factors, oxidative stress, and brain damage markers were detected through ELISA. The expression of apoptosis-related proteins and PI3K/PDK1/AKT signaling pathway related proteins were measured by western blot. The expressions of PI3K, PDK1, AKT, and MBP in paraventricular white matter cells were detected by immunofluorescence.

**Results:**

Naomaitai treatment decreased neurological function score in rats with vascular dementia, ameliorated paraventricular white matter damage caused by long-term hypoxia, and hypoperfusion reduced the brain injury markers S-100*β* and NSE contents, suppressed inflammatory reaction and oxidative stress, reduced IL-1*β*, IL-6, TNF-*α*, and MDA contents, and remarkably increased IL-10 and SOD contents. TUNEL and western blot assay showed that Naomaitai treatment decreased neuronal cell apoptosis, increased Bcl-2 expression, and reduced caspase-3 and Bax expression. Furthermore, we found Naomaitai inhibited PI3K and PDK1 expression and activated phosphorylated AKT protein in rats with vascular dementia. However, the protective effect of Naomatai in rats with vascular dementia was inhibited, and expression of PI3K signaling pathway-related proteins was blocked after administration of PI3K inhibitor.

**Conclusion:**

Naomaitai can ameliorate brain damage in rats with vascular dementia, inhibit neuronal apoptosis, and have anti-inflammatory and antioxidative stress effects, which may be regulated by the PI3K/PDK1/AKT signaling pathway.

## 1. Introduction

Vascular dementia (VD) is a severe cognitive impairment syndrome caused by long-term hypoxia and hypoperfusion in the brain [1]. Cerebrovascular diseases, such as ischemic stroke, hemorrhagic stroke, and cerebral ischemia and hypoxia, are the main clinical causes for VD [2]. The aging of the social population makes VD a recognized public health issue [3]. VD is the second largest type of dementia after Alzheimer's disease. The research showed that long-term hypoxia and hypoperfusion in the brain region caused by the damage to the paraventricular white matter is the main factor of VD [4]. Therefore, white matter injury is also a unique clinical diagnostic basis for VD. The damaged areas showed pathological changes such as glial cell activation and proliferation [5]. Glial cell activation produced reactive oxygen species, which promoted the occurrence of oxidative stress [6]. In addition, cytokine secretion also stimulated inflammatory reaction and aggravated white matter damage [7].

Naomaitai (NMT) is a new type of traditional Chinese medicine developed by Guilin Sanjin Pharmaceutical Co., Ltd. NMT is composed of Sanchi, ginkgo leaf, Safflower and Danshen Root, etc. NMT can benefit vital energy, promote blood flow, stop endogenous wind, and reduce phlegm [8]. In the clinic, NMT is commonly used in the recovery period of ischemic stroke (cerebral infarction) [9–11]. Chinese medicine syndrome belongs to the meridian qi deficiency and blood stasis and wind phlegm and blood stasis to obstruct the arteries and veins. It has been proved that cerebral ischemia and hypoxia are the main cause of VD [5]. NMT can repair hypoxia and hypoperfusion and cognitive impairment in the rats' model of VD. NMT can play a protective role in the white matter of VD rats by improving the blood circulation of the brain [8]. And several studies have shown that most of the components of NMT have an effect on cerebral ischemia and hypoxia [12–19]. But no literature has reported the specific mechanism of the protective effect of NMT. This study explored the protective effect and mechanism of NMT on neurons and white matter in VD rat models, which may be associated with the PI3K/PDK1/AKT signaling pathway.

## 2. Materials and Methods

### 2.1. Experimental Animals and Group Assignment

Fifty specific-pathogen-free male adult Sprague-Dawley rats weighing 260-300 g were purchased from the Department of Experimental Animals, China Medical University, China. This study was approved by the Laboratory Animal Welfare and Ethics Committee, China Medical University (IACUC NO. 2015047). The rats were randomly assigned to sham group (sham-operated group, n = 10), VD group (n = 10), HNMT group (VD + high-dose NMT; n = 10), LNMT group (VD + low-dose NMT; n = 10), and PI3K group (VD + high-dose NMT + PI3K inhibitor LY294002; n = 10). 

### 2.2. Model Preparation

The rats were fasted but allowed free access to water 12 hours before surgery. The rats were anesthetized with 2% sodium pentobarbital 0.3 ml/100 g until rat's limbs were soft and the conjunctival reflex disappeared. The rats were fixed on the board in the supine position. After shaving on the neck and sterilizing with alcohol cotton, a median incision (approximately 1 cm) was made to bluntly dissociate fascia and subcutaneous tissue. The common carotid artery is located below the sternocleidomastoid muscle. The bilateral common carotid artery and vagus nerve were gently separated using a glass minute hand soaked in PBS buffer. The common carotid artery was permanently ligated at the distal end with a No. 4 surgical line. After the surgery, two drops of penicillin were dropped into the wound to prevent infection. The skin was sutured and sterilized with physiological saline. In the PI3K group, PI3K inhibitor LY294002 solution was administered 20 minutes before VD establishment. The operation in the sham group was identical to that mentioned above, without ligation. All rats maintained a body temperature of 37°C during anesthesia. After consciousness, the rats were housed at room temperature. Penicillin was intraperitoneally injected for two consecutive days to prevent infection.

Six weeks after surgery, the behavioral symptoms of rats were observed, neurological function scores were performed, and the cognitive ability and memory ability of each group were evaluated by water maze. After the experiment, the blood was collected by Orbital Blood collection and centrifuged to get serum. The brain tissue of each group was taken out. Half of the brain tissue was fixed in 4% paraformaldehyde, and the other half of the brain tissue was removed. To obtain the white matter, the rat's brain was cut into 2mm thick cerebral coronal slices. A thin white structure between the cortex and the deep brain tissue (such as the hippocampus) was observed, which was the cerebral white matter, then separated with a scalpel, cooled on the ice, and stored at -80°C.

### 2.3. Dosage and Method of Administration

In the PI3K group, after anesthesia, the head was fixed with a stereotactic instrument. Stereotaxic location on the lateral ventricle: a hole was drilled with a dental drill 0.9 mm lateral and 1.5 mm posterior to Bregma, at the depth of 3.8 mm. 10 *μ*l 10 mmol/L LY294002 solution was slowly injected with a microsyringe within 5 minutes. The microsyringe was maintained in place for 5 minutes and then slowly withdrawn. Make sure to observe that there is no blood, no liquid medicine, or no cerebrospinal fluid overflow at the puncture point after needle extraction, the scalp was sutured, and the puncture was completed. In the LNMT and HNMT groups, according to previous experimental animal studies, the rats were intragastrically administered 0.56g/kg and 2.24g/kg NMT, respectively [20]. The same batch (Batch No. 2018010) of Naomaitai Capsule was used in this study to ensure a stable therapeutic effect. Naomaitai powder was dissolved in double distilled water and heated in a water bath at 37°C, vortex to a suspension of powder evenly dispersed. The rats in the PI3K group were treated with the dose of HNMT group. NMT administrate once 2mL a day for 5 days and operation 2 days after discontinuation of medication. In the model and sham groups, the equivalent amount of double distilled water was used as a placebo during the treatment.

### 2.4. Neurological Deficit Score

Bederson scoring system was used to detect neurological impairment in rats. At 6 weeks after surgery, a single blind method was used to score neurological symptoms (that is, observers did not know the way of group assignment). Using the Bederson neurological deficit score method as a standard, the symptoms of each group of rats were observed; the neurological function of the rats was scored; and the symptoms of neurological deficit were recorded [21]. The scoring criteria are as follows: score 0: no symptoms of nerve damage; score 1: the contralateral forelimb cannot be fully extended when lifting the tail; score 2: turn to the temporal side while walking; score 3: fall to the opposite side of the lesion; score 4: cannot walk spontaneously.

### 2.5. Morris Water Maze Test

The Morris water maze consists of a circular pool of 1.5 m diameter, a camera directly above the pool, and a computer program connected to the camera. Forced swimming: The navigation test was conducted to observe the time spent in the target quadrant for 5 days of training. After 5 days of training, the platform was removed from the water. The spatial probe test was performed to observe the number of platform location crosses so as to measure cognitive and memory abilities. At 6 weeks after surgery, Morris water maze test was carried out to assess the cognitive and memory abilities in VD rats.

### 2.6. HE Staining

At 6 weeks after model establishment, five rats from each group were obtained for HE staining in rats; the samples were obtained. After decapitation, the white matter was gained, fixed in 10% paraformaldehyde for 48 hours, dehydrated, permeabilized, embedded in wax, and sliced into sections. The sections were stained with hematoxylin and eosin. The histopathological changes of cerebral white matter were observed under a light microscope.

### 2.7. TUNEL Assay for Neuronal Cells in the White Matter

TUNEL Kit (No. 11684817910, Roche, USA) was used to detect the apoptosis of white matter neurons according to the detailed instructions. The white matter fixed in paraformaldehyde was dehydrated, embedded, and sliced. The sections were incubated with 0.9% NaCl for 5 minutes and washed twice with PBS. After pouring out the liquid, biotinylated nucleotides and terminal deoxynucleotidyl transferase (1:500 dilution) were added and mixed well. After covering plastic cover glass, the mixture was incubated for 60 minutes at 37°C, washed with PBS, and treated with 0.3% H_2_O_2_. After three washes with PBS, horseradish peroxidase- (HRP-) labeled streptavidin was added and incubated for 30 minutes at room temperature, followed by PBS washes. The sample was observed and photographed with a microscope.

### 2.8. ELISA for MDA and SOD Contents as Well as S-100*β* and NSE Contents in Brain

Oxidative stress factor and brain damage markers contents in white matter were determined by ELISA. ELISA kit was used to detect the contents of white matter oxidative stress factors MDA (CEA597Ge, uscn, USA) and SOD (SES134Ra, uscn, USA) as well as white matter damage markers S-100*β* (SEA567Ra, uscn, USA) and NSE (SEA537Ra, Uscn, USA) contents according to the instructions. 100 ul standard preparation and l00 ul diluted sample were added to the corresponding reaction plate well, mixed, and incubated at 37°C for 30 minutes. After the plate was washed, 100 ul tested sample was added in each well and incubated for 2 hours at 37°C. After the plate was washed, l00 ul HRP-labeled secondary antibody was added and incubated for 30 minutes at 37°C. After the plate was washed, the samples were visualized by adding 50 ul coloring solution A and coloring solution B in the dark for 15 minutes. The reaction was terminated by adding 50 ul stop buffer. Optical density (OD) values were read at 450 nm using the microplate reader (EXL808, USA). The standard curves were drawn. The corresponding concentration of the sample was calculated according to the curve equation.

### 2.9. Western Blot Assay to Detect Related Protein Expression

The protein expression of PI3K/PDK1/AKT signaling pathway-related proteins, apoptosis-related proteins were detected by Western blot.

Through after frozen white matter was added to the precooling tissue protein lysate, homogenate suspension was made by tissue homogenizer. Protein content was detected in BCA protein detection kit. The protein concentration was adjusted for SDS-polyacrylamide gel electrophoresis, and the proteins were transferred onto the membrane using a Trans-Blot transfer system (1703930, BIO-RAD, USA). The membrane was blocked with confining liquid for 2 hours. PI3K (ab151549, Abcam, USA), PDK1 (ab110025, Abcam, USA), AKT (ab8805, Abcam, USA), Bcl-2 (ab59348, Abcam, USA), Caspase3 (ab13847, Abcam, USA), and Bax (ab32503,Abcam, USA) primary antibodies were added and incubated at 4°C overnight. After three washes with TBST, secondary antibodies were added and incubated for 1 hour, followed by four washes with TBST. The sample was visualized with ECL luminescent kit (35050, Pierce, USA) and imaged with the gel imaging system. The gray value was analyzed by Quantity One software.

### 2.10. Detection of White Matter Nerve Fiber Damage by LFB Staining

The brain tissue sections of each group were taken. Three sections were taken from each rat. After dewaxing, the sections were put into LFB staining solution, sealed and immersed for 24 hours at 60°C, Washed with distilled water and 95% ethanol. After separating the color with a 0.05% lithium carbonate aqueous solution, the color separation was continued with 70% alcohol until the gray and white matter were clearly observed under the microscope. The gray matter is transparent and the white matter is blue. After being dehydrated by alcohol gradient, samples were transparent twice by xylene and sealed by the neutral gum was. The damage of the white matter nerve fibers was observed under microscope.

### 2.11. Immunofluorescence for the Expression of PI3K, PDK1, AKT and MBP at the White Matter

Paraffin sections were dewaxed, hydrated, immersed in 3% hydrogen peroxide solution for 15 minutes, and washed with PBS. Antigens were retrieved with 0.1 M sodium citrate. The sections were blocked with goat serum and incubated for 30 minutes at 37°C. After removal of serum, without washing, PI3K (ab151549, Abcam, USA), PDK1 (ab110025, Abcam, USA), AKT (ab8805, Abcam, USA), and MBP (ab40390, Abcam, USA) antibodies were added and incubated at 4°C overnight. The sections were washed with PBS and incubated with fluorescence-labeled secondary antibody for 30 minutes at 37°C, followed by PBS washes. DAPI-stained nuclei were added and incubated at room temperature for 10 minutes, followed by PBS washes. The sections were mounted with neutral resin and observed with a fluorescence microscope.

### 2.12. Statistical Analysis

Each part of the results was derived from 3 independent replicates of experiment. Data were analyzed using SPSS 19.0 software and expressed as the mean ± standard deviation. Intergroup comparison was carried out with one-way analysis of variance. Intragroup comparison was conducted using repeated measures analysis of variance. A value of* P* < 0.05 was considered statistically significant.

## 3. Results

### 3.1. NMT* Ameliorated* Neurological Impairment in VD Rats

**Figure 1:**
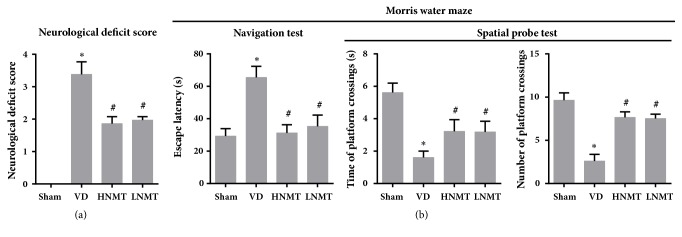
NMT lessens neurological impairment in VD rats. Bederson scoring system was used to detect neurological impairment in rats. MWM test was conducted to determine the spatial cognition and memory ability of rats. (a) The neurological deficit score; (b) Morris water maze test. *∗*P<0.05 vs. Sham group; #P<0.05 vs. VD group.

Afterbilateral common carotid arteryocclusion, compared with the sham group (0±0) in the VD group the neurological deficit score increased (3.38±0.39) and spatial cognitive ability and spatial memory ability were poor (*P *< 0.05). After NMT treatment, the neurological deficit score decreased and spatial cognitive ability and spatial memory ability were effectively improved in VD rats. Nevertheless, neurological deficit score results were not significantly different between the HNMT (1.86±0.22) and the LNMT (1.97±0.11) groups (Figure 1(a)).

After 5 days of training, it demonstrated that the latency in the navigation test was long in VD rats. In the spatial probe test, VD rats showed nondirectional trajectory in the pool. Compared with the sham group (the time spent in the target quadrant, 5.62±0.58; the number of platform location crosses, 9.65±0.84), the time in the target quadrant (1.61±0.49) and the number of platform location crosses (2.6±0.77) were significantly reduced (*P *< 0.05). After NMT treatment, rats swam in the pool under the same conditions in the HNMT and LNMT groups. The navigation test and spatial probe test results demonstrated that the latency finding the platform was remarkably shortened, and the behavior of the rats was purposeful after removing the platform. The time in the target quadrant (HNMT, 3.22±0.72; LNMT, 3.18±0.66) and the number of platform location crosses (HNMT, 7.66±0.63; LNMT, 7.52±0.51) were obviously increased (vs. VD,* P *< 0.05) (Figure 1(b)). The results indicated that NMT has a therapeutic effect on neurological deficit rats, and the therapeutic effect is not associated with the dosage.

### 3.2. NMT Improves White Matter Damage in VD Rats

**Figure 2:**
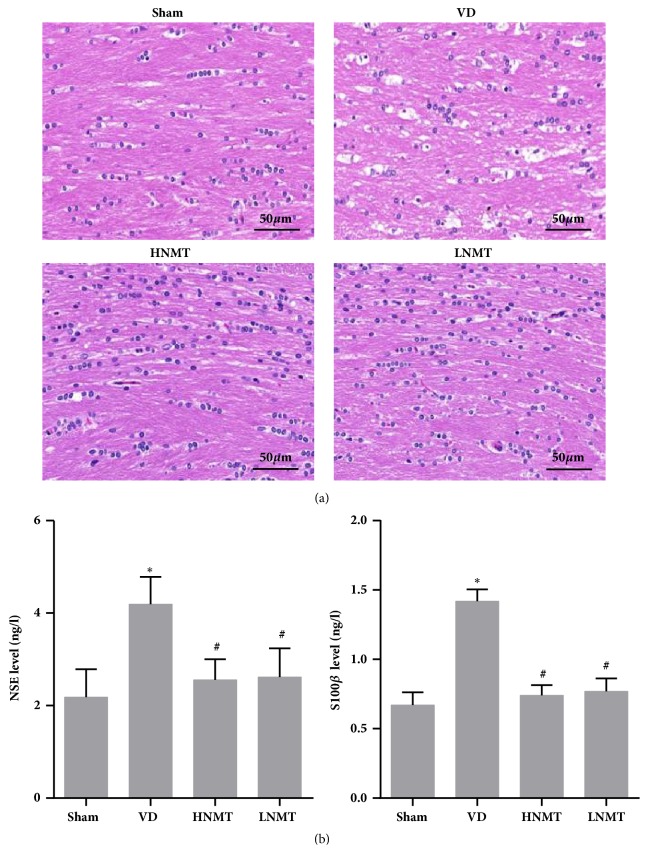
NMT improves white matter damage in VD rats. HE staining was utilized to observe the changes of brain tissue morphology in rats. ELISA was performed to determine the expression levels of brain damage markers. (a) Hematoxylin & eosin staining (Bar, 50*μ*m); (b) ELISA was performed to determine the expression levels of NSE and S-100*β*. *∗*P<0.05 vs. Sham group; #P<0.05 vs. VD group.

HE staining result showed that the structure of brain was normal, the fibers of the corpus callosum were arranged and there was no obvious vacuolar structure in the Sham group. In the VD group, the white matter was damaged, the fibers of the corpus callosum were disordered, and a large number of vacuolar structures were observed. Elisa showed that S-100*β* and NSE contents markedly increased in the VD group (S-100*β*, 1.42±0.09; NSE, 4.19±0.59;* P *< 0.05) compared with the sham group (S-100*β*, 0.67±0.09; NSE, 2.18±0.61). In the HNMT and LNMT groups, the fibers of the corpus callosum are arranged and only a few vacuoles appeared in the white matter. S-100*β* (HNMT, 0.74±0.07; LNMT, 0.77±0.09) and NSE (HNMT, 2.56±0.44; LNMT, 2.62±0.62) contents diminished (vs. VD,* P *< 0.05). There were no significant differences between the HNMT and the LNMT groups (Figure 2). These findings indicated that NMT can repair the white matter damage in VD rats.

### 3.3. NMT Suppresses Oxidative Stress in the White Matter of VD Rats

**Figure 3:**
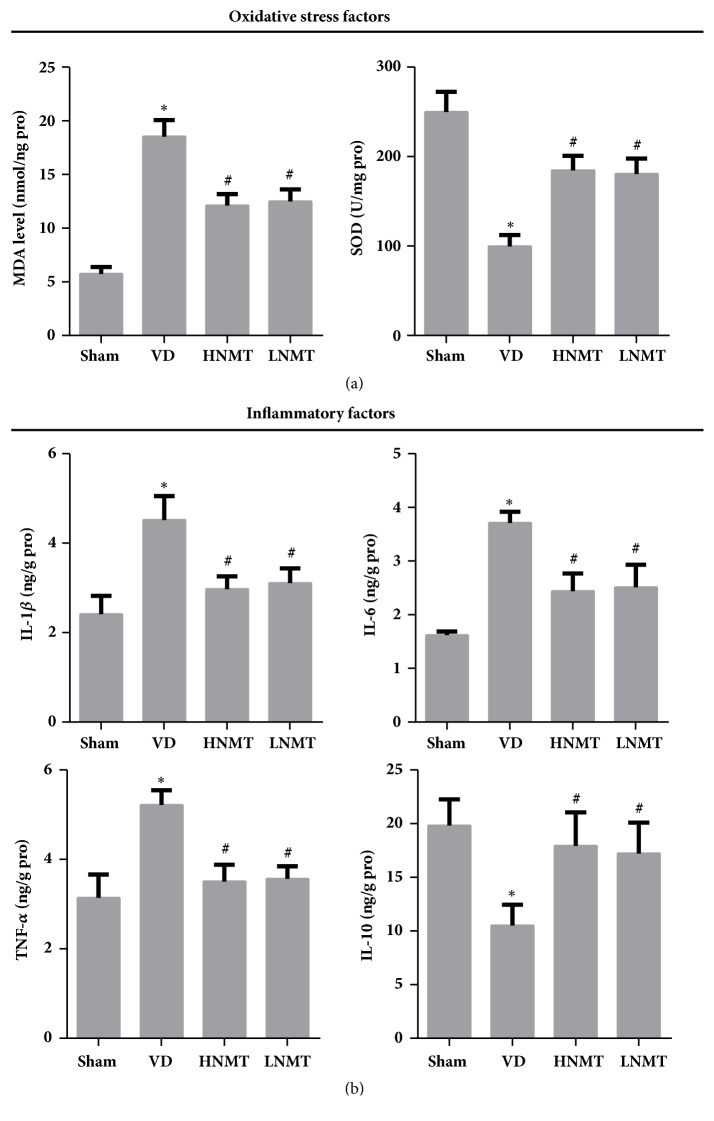
NMT suppresses oxidative stress and inflammation in the white matter of VD rats. ELISA was performed to determine the expression levels of oxidative stress factors and inflammation related factors contents in white matter. (a) The expression levels of oxidative stress factors (MDA and SOD) in white matter; (b) the expression levels of inflammation related factors (IL-1*β*, IL-6, TNF-*α* and IL-10) contents in white matter. *∗*P<0.05 vs. Sham group; #P<0.05 vs. VD group.

After hypoxia/hypoperfusion in the brain, the content of MDA in white matter significantly increased, while the content of SOD in white matter decreased in VD rats (MDA, 18.53±1.55; SOD, 99.74±12.53;* P *< 0.05) compared with the sham group (MDA, 5.73±0.66; SOD, 249.86±22.51). After intragastric administration of NMT, MDA (HNMT, 12.11±1.06; LNMT, 12.49±1.13) content reduced, but SOD (HNMT, 184.62±16.22; LNMT, 180.55±17.41) content increased in the white matter in the HNMT and LNMT groups (vs. VD,* P *< 0.05) (Figure 3(a)). These results indicated that NMT inhibits white matter oxidative stress and improves white matter damage.

### 3.4. NMT Suppresses White Matter Inflammation Induced by Chronic Hypoxia and Hypoperfusion in Rats

ELISA showed that IL-1*β*, IL-6, and TNF-*α* expression dramatically increased compared with the sham group (IL-1*β*, 2.41±0.41; IL-6, 1.62±0.09; TNF-*α*, 3.14±0.52; IL-10, 19.82±2.44), but IL-10 expression reduced in the VD group (IL-1*β*, 4.52±0.53; IL-6, 3.71±0.21; TNF-*α*, 5.22±0.33; IL-10, 10.51±1.93;* P *< 0.05). The results suggested that continuous hypoxia and hypoperfusion of the brain triggered inflammation in the cerebral white matter. IL-1*β*, IL-6, and TNF-*α* expression significantly diminished, but IL-10 expression significantly increased in the white matter in the HNMT (IL-1*β*, 2.97±0.29; IL-6, 2.44±0.23; TNF-*α*, 3.51±0.37; IL-10, 17.93±3.12) and LNMT (IL-1*β*, 3.11±0.33; IL-6, 2.51±0.42; TNF-*α*, 3.57±0.28; IL-10, 17.22±2.87) groups (vs. VD,* P *< 0.05) (Figure 3(b)). These findings confirmed that NMT achieves the therapeutic effect by inhibiting the expression of inflammatory factors.

### 3.5. Naomaitai Can Reduce Myelin Demyelination and Improve White Matter Damage in VD Rats

**Figure 4:**
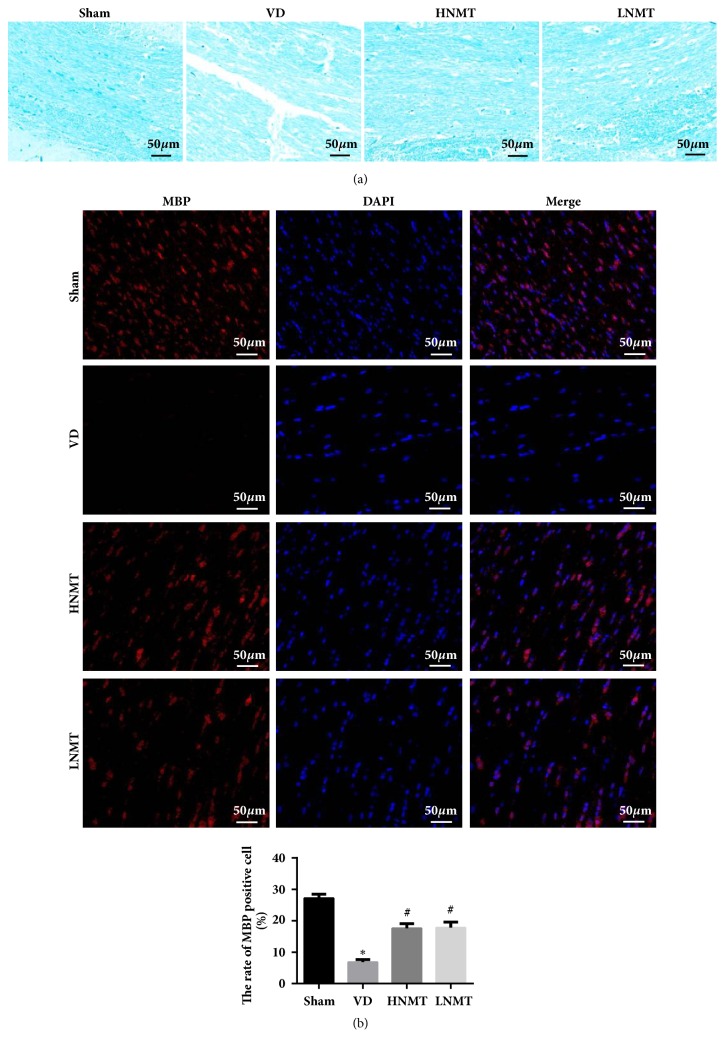
Naomaitai can reduce myelin demyelination and improve white matter damage in VD rats. LFB staining was utilized to observe the damage of white matter nerve fibers and the loss of myelin sheath. Immunofluorescence method was used to determine the MBP expression in white matter of rats. (a) LFB staining (bar, 50*μ*m); (b) the expression of MBP determined by Immunofluorescence method (bar, 50*μ*m). *∗*P<0.05 vs. Sham group; #P<0.05 vs. VD group.

To observe the damage of white matter nerve fibers and the loss of myelin sheath, LFB staining was performed. It showed in sham group the myelin sheath fibers in the corpus callosum were arranged neatly and compactly, oligodendrocyte structure was intact, no vacuole structure. In VD group, the gap between myelin sheaths became larger, and a large number of vacuoles were observed due to oligodendrocyte decreasing, Myelin arrangement is irregular, more chaotic, and the staining is obviously lighter (vs. sham), which were typical demyelinating lesions appeared in corpus callosum. After administration of Naomaitai, the myelin sheath in LNMT group was denser and the disordered arrangement was improved. The staining degree of LFB was significantly higher than that in VD group. There was no significant difference between HNMT group and LNMT group (Figure 4(a)).

MBP is the basic protein in myelin sheath. The MBP in white matter of rats in each group was observed by IF. The results showed that the MBP in the sham operation group was higher. However, in VD group, white matter damage was caused by chronic white matter deficiency, and MBP expression decreased (vs. sham, P<0.05). After Naomaitai administrated, the expression of MBP in white matter of rats in HNMT group and LNMT group increased (vs. VD, P < 0.01). There was no significant difference in protein expression between the two treatment groups (P>0.05) (Figure 4(b)). The results suggested that Naomaitai can improve the low expression of MBP caused by myelin sheath injury, reduce the damage and loss of myelin sheath caused by insufficient cerebral perfusion, and play a protective role in white matter.

### 3.6. NMT Inhibits Apoptotic Factor Expression in the White Matter of VD Rats

**Figure 5:**
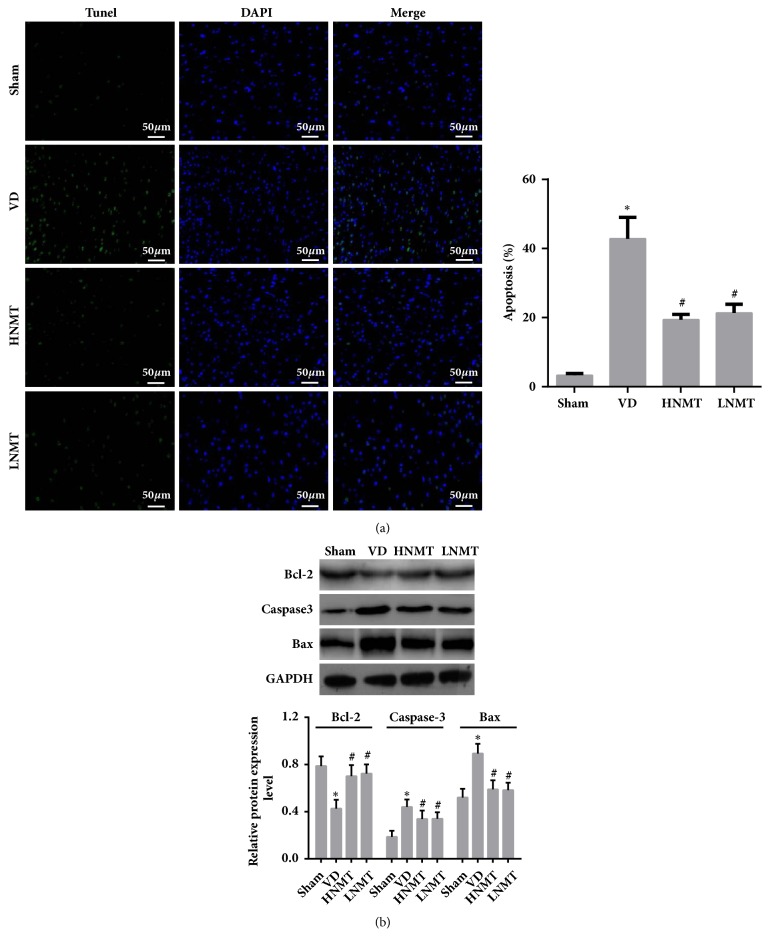
NMT inhibits apoptotic factor expression in the white matter of VD rats. Apoptosis-positive cells were determined via Tunel assays. Apoptosis-related protein expression was determined via Western blot assay. (a) Tunel assays (bar, 50*μ*m); (b) Bcl-2, Caspase-3 and Bax protein expression determined via Western blot assay. *∗*P<0.05 vs. Sham group; #P<0.05 vs. VD group.

TUNEL assay (Figure 5(a)) and western blot assay (Figure 5(b)) showed that white matter neurons had obvious apoptosis compared with sham group (apoptotic rate, 3.31±0.52), and apoptotic rate remarkably increased in the VD group (apoptotic rate, 42.82±6.22). Apoptosis-related protein expression data demonstrated that Bcl-2 expression reduced, caspase-3 and Bax expression increased in the VD group (vs. Sham,* P *< 0.05). The apoptotic rate decreased; but Bcl-2 expression increased; and caspase-3 and Bax expression reduced in the HNMT (apoptotic rate, 19.42±1.49) and LNMT (apoptotic rate, 21.35±2.52) groups (vs. VD,* P *< 0.05). The results suggested that NMT plays an antiapoptotic role in the VD model.

### 3.7. NMT Mitigates Brain Injury in VD Rats through the PI3K/PDK1/AKT Signaling Pathway

**Figure 6:**
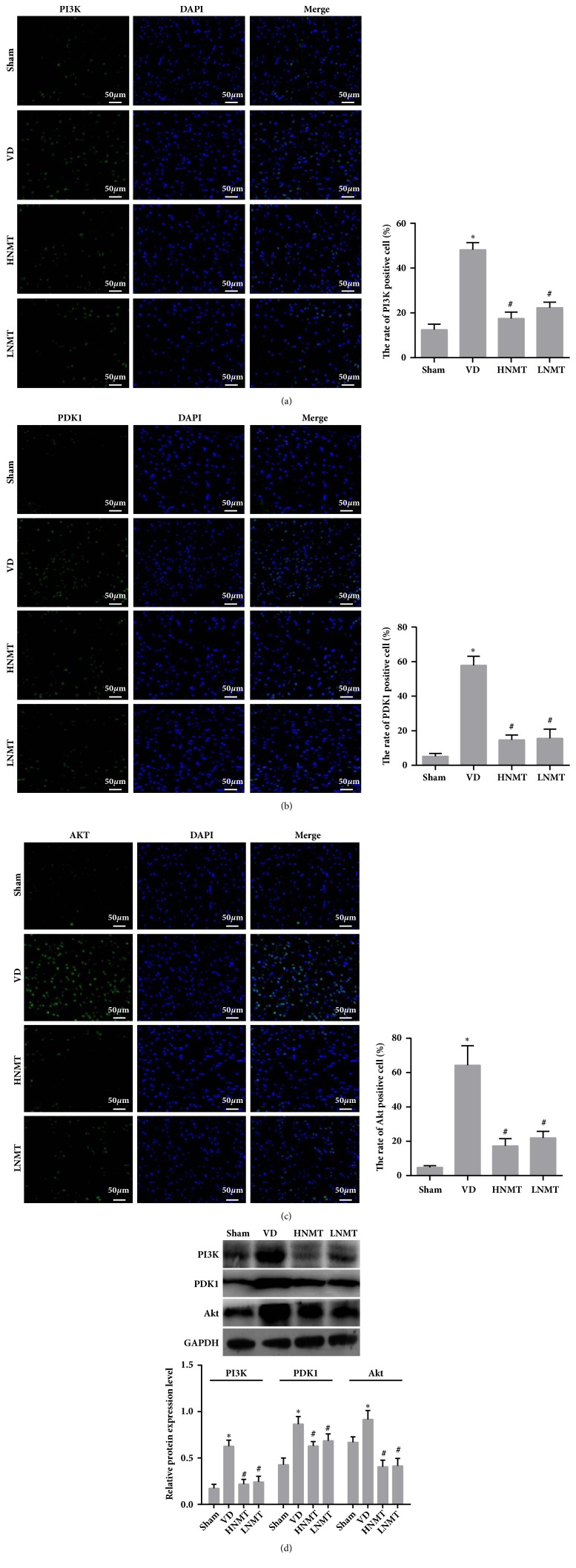
NMT mitigates brain injury in VD rats through the PI3K/PDK1/AKT signaling pathway. Immunofluorescence method and western blot assay were used to determine the protein expression of PI3K, PDK1, and AKT. (a) The protein expression of PI3K determined by Immunofluorescence method (bar, 50*μ*m); (b) the protein expression of PDK1 determined by Immunofluorescence method (bar, 50*μ*m); (c) the protein expression of Akt determined by Immunofluorescence method (bar, 50*μ*m); (d) the protein expression of PI3K, PDK1, and AKT determined by Western blot assay. *∗*P<0.05 vs. Sham group; #P<0.05 vs. VD group.

Immunofluorescence method (Figures 6(a)–6(c)) and western blot assay (Figure 6(d)) were used to determine the protein expression of PI3K, PDK1, and AKT. The results revealed that PI3K, PDK1, and AKT expression increased in the white matter after model establishment in the VD group (vs. Sham,* P *< 0.05). After NMT treatment, PI3K, PDK1, and AKT expression decreased in the white matter in the HNMT and LNMT groups (vs. VD,* P *< 0.05). Our results indicated that NMT could ameliorate hypoxia and ischemia in the white matter of VD rats, and the therapeutic effect may be associated with the inhibition of expression of the PI3K/PDK1/AKT signaling pathway.

### 3.8. PI3K Inhibitor Reverses the Therapeutic Effect of NMT on VD Rats

**Figure 7:**
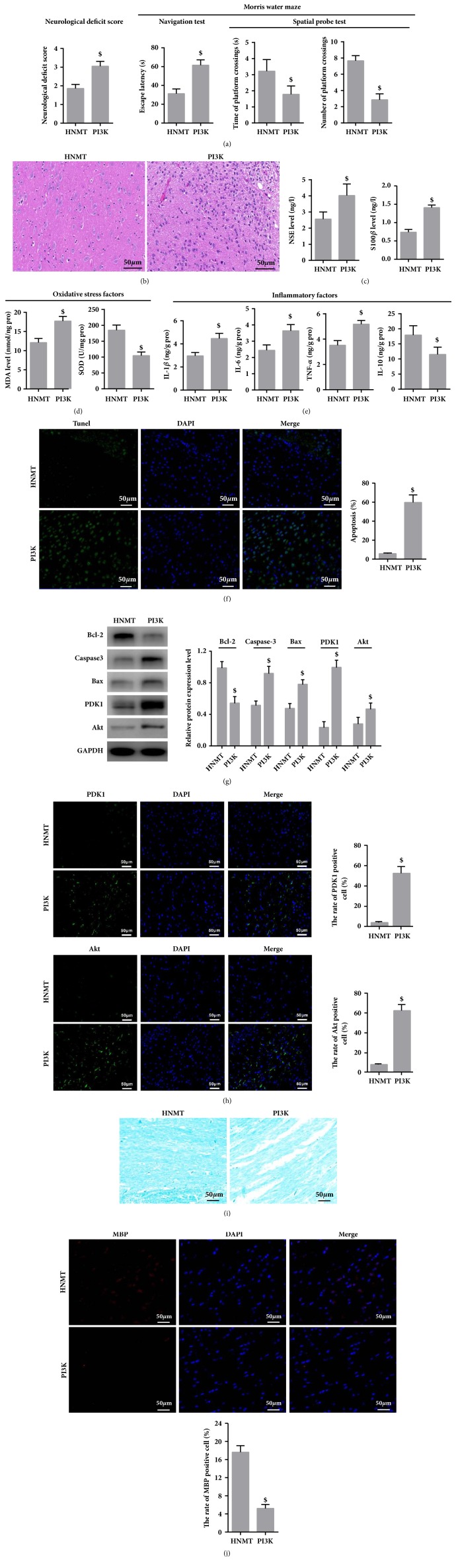
PI3K inhibitor reverses the therapeutic effect of NMT on VD rats. Bederson scoring system was used to detect neurological impairment in rats. MWM test was conducted to determine the spatial cognition and memory ability of rats. HE staining was utilized to observe the changes of brain tissue morphology in rats. ELISA was performed to determine the expression levels of brain damage markers, oxidative stress factors and inflammation related factors. Apoptosis-positive cells were determined via Tunel assays. Apoptosis-related protein expression was determined via Western blot assay. Immunofluorescence method and western blot assay were used to determine the protein expression of PI3K, PDK1, and AKT. LFB staining was utilized to observe the damage of white matter nerve fibers and the loss of myelin sheath. Immunofluorescence method was used to determine the MBP expression in white matter of rats. (a) The neurological deficit score and Morris water maze test; (b) hematoxylin & eosin staining (bar, 50*μ*m); (c) ELISA was performed to determine the expression levels of NSE and S-100*β*; (d) the expression levels of oxidative stress factors (MDA and SOD) in white matter; (e) the expression levels of inflammation related factors (IL-1*β*, IL-6, TNF-*α*, and IL-10) contents in white matter; (f) Tunel assays (bar, 50*μ*m); (g) the protein expression of PI3K, PDK1 and AKT determined by Western blot assay; (h) the protein expression of PDK1 and Akt determined by Immunofluorescence method (bar, 50*μ*m); (i) LFB staining (bar, 50*μ*m); (j) the expression of MBP determined by Immunofluorescence method (bar, 50*μ*m). $P<0.05 vs. HNMT group.

The results showed that neurological deficit score increased, spatial cognitive ability and spatial memory ability were poor in the PI3K group (neurological deficit score, 3.05±0.26; the time spent in the target quadrant, 1.78±0.52; the number of platform location crosses, 2.85±0.74) (vs. HNMT,* P *< 0.05) (Figure 7(a)). Hematoxylin-eosin staining results demonstrated that neuronal damage was obvious and vacuolar structure increased in the white matter in the PI3K group (vs. HNMT,* P *< 0.05) (Figure 7(b)). ELISA revealed that S-100*β* (1.41±0.07), IL-1*β* (4.47±0.45), IL-6 (3.64±0.38), TNF-*α* (5.18±0.29), MDA (17.72±1.23), and NSE (1.78±0.52) contents increased but IL-10 (11.52±2.41) and SOD (104.49±11.52) contents decreased in the white matter (vs. HNMT,* P *< 0.05) (Figures 7(c)–7(e)). The results suggested that PI3K inhibitors depressed the protective effect of NMT on the cerebral white matter. Furthermore, Tunnel assay and results indicated that apoptotic rate (59.72±7.93) of neurons increased in the white matter in the PI3K group (vs. HNMT,* P *< 0.05) (Figure 7(f)). Western blot assay indicated that Bcl-2 expression matter decreased and the expression of caspase-3 and Bax increased in the white matter in the PI3K group (vs. HNMT,* P *< 0.05) (Figure 7(g)). Western blot assay (Figure 7(g)) and immunofluorescence method (Figure 7(h)) were conducted to measure PDK1 and AKT expression in the PI3K group. Our results showed that expression of related proteins significantly increased (vs. HNMT,* P *< 0.05). LFB staining showed that there were demyelinating lesions in the corpus callosum of rats in PI3K group. The structure of myelin sheath was loose, and a large number of oligodendrocytes were lost and formed vacuoles. The myelin sheath was disordered and stained shallowly (PI3K vs. HNMT) (Figure 7(i)). IF results showed that the MBP in PI3K group was upregulated (vs. HNMT) (Figure 7(j)).

## 4. Discussion

This study identified that NMT had a significant effect on improving neurological impairment and alleviating ischemia and hypoxia in VD rats. Simultaneously, NMT could also achieve the therapeutic effect of inhibiting oxidative stress, reducing white matter-related apoptosis protein and PI3K/PDK1/AKT signaling pathway protein expression. These results suggested that NMT has protective effects on white matter of VD rats, which may be achieved by regulating PI3K/PDK1/AKT signaling pathway.

VD is a common disease in the elderly population, and is an age-related cognitive disorder [22]. A large amount of data showed that most patients with dementia presented different degrees of white matter damage [23]. We infer that the key factors affecting cognitive ability are white matter damage, inflammation in the central nervous system, oxidative stress and neuronal apoptosis caused by long-term hypoxia and hypoperfusion, which are also the main causes of white matter damage. Therefore, strengthening the protection of white matter and reducing neuronal apoptosis are key issues in the treatment of VD [24].

Excitatory amino acid toxicity, free radicals and NO-induced oxidative stress injury and inflammatory reaction are the main factors that mediate neuronal apoptosis after cerebral ischemia [25–27]. Apoptosis is the phenomenon of programmed cell death under gene regulation [28]. Bcl-2 encoded by Bcl-2 proto oncogene plays an important role in promoting cell survival and apoptosis [29]. Activation of a series of downstream genes of pro-apoptotic factor Bax can accelerate cell apoptosis [30]. Caspase is an aspartate-specific protease and a major performer of apoptosis [31]. Caspase-3 is the most important apoptotic factor in the caspase apoptotic pathway [32]. PI3K/PDK1/AKT signaling pathway is the key signal transduction pathway to inhibit cell apoptosis in cells [33]. Because of PI3K activation stimulated by external stimuli, phosphatidylinositol phosphorylation is catalyzed to produce the second messenger: inositol [34]. Under the control of inositol, AKT is transferred from cytoplasm to cell membrane, and is phosphorylated by PDK1 assisted AKT protein threonine site [35]. The active AKT after phosphorylation regulates the related expression of downstream apoptotic factors and inhibits apoptosis [36].

Previous studies have found that many traditional Chinese medicines have therapeutically effect on VD [37–40]. Zhu et al. have found that Panax ginseng extract is neuroprotective against vascular dementia induced by chronic cerebral hypoperfusion [37]. Fan et al. have identified that moxibustion intervention is able to improve the delayed memory in VD rats [38]. NMT capsule takes ginseng and Sanchi as principal drugs, which can invigorate vital energy, produce blood, dissipate blood stasis, and promote blood circulation [41]. NMT capsule takes Chinese Angelica, ginkgo leaf, and Danshen Root as ministerial drugs, which can clear and activate the channels and collaterals [8]. As the remarkable effects of supplementing* Qi*, activating blood circulation, removing blood stasis, dredging collaterals, expelling wind, and eliminating phlegm, NMT capsule is clinically used in the recovery phase of ischemic stroke [10, 11]. Previous studies showed that NMT has therapeutic effect on VD [20, 42, 43] and the specific mechanism of the protective effect has not been reported. Ischemic stroke is equivalent to acute ischemic cerebrovascular disease such as cerebral infarction in western medicine [44]. Therefore, we can conclude that NMT capsule can improve cerebral hypoperfusion in the brain of rats. NMT has been shown to reduce the occurrence of cognitive impairment by improving the cerebral ischemia and hypoxia in VD rats and protecting the white matter [8].

It was shown that, after bilateral common carotid arteryocclusion 6 weeks, rats not only showed behavioral disorders but long-term hypoperfusion activated cells in the white matter, which in turn led to proliferative lesions, enlarged cell bodies, and many neurites and mediated the inflammatory response by secreting a series of active substances [45]. White matter damage caused by long-term hypoxia and hypoperfusion can cause cognitive decline, oxidative stress during cerebral ischemia, and excessive free radical [46]. Although there are few studies on the detection of oxidative stress markers in VD, some data support the existence of oxidative stress in VD [47, 48]. Sinclair et al. [49] pointed out that the level of antioxidant trace element vitamin C decreased in plasma of VD. Furthermore, Casado et al. [50] proved that malondialdehyde levels were higher in VD patients compared with the control group. 8-Hydroxy-2 deoxyguanosine (8-OHDG) contents obviously increased in the cerebrospinal fluid and urine of VD patients [51]. These studies showed that oxidative stress levels are altered in VD. Cerebral autosomal dominant arteriopathy with subcortical infarcts and leukoencephalopathy(CADASIL) is a type of VD [52]. The studies have found that there is extensive neuronal apoptosis in the brain of CADASIL patients, and the occurrence of apoptosis is due to hypoxia and hypoperfusion injury in the brain [53]. Further research on the apoptotic pathway is expected to provide new treatments for VD. Therefore, this study examined inflammatory factors, oxidative stress factors and apoptotic factors in VD model rats. The results showed that NMT can inhibit the occurrence of inflammatory response, oxidative stress and apoptosis. The results suggested that the protective effect of NMT may be associated with its inhibition of white matter inflammatory reaction, peroxidation and apoptosis.

The PI3K/PDK1/AKT signaling pathway plays an important role in the regulation of cell proliferation, and its main signaling molecules include PI3K, PDK1 and AKT [54]. Activation of PI3K activates downstream protein kinases, PDK1, and AKT and both PDK1 and Akt contain a PH domain [55]. This domain binds to PI(3,4,5)P3, translocating them to the plasma membrane of the cell and phosphorylation of PDKI activates AKT. Activated AKT phosphorylates Bax and inhibits the formation of dimers between Bax and Bcl-2 [56]. Dissociated Bcl-2 plays a key role in inhibiting apoptosis [57]. PDK1 is a downstream protein of PI3K and plays an important role in physiological processes such as cell metabolism, growth and survival [58]. Cellular assay results show that PDK1 loss leads to a large number of apoptosis; and a major network composed of Cyclin, cyclin-dependent protein kinase (CDK), and CDK inhibitory protein (CKIs) can coordinate the regulation of the cell cycle [59]. In the present study, a VD rat model was established and was given different doses of NMT simultaneously. Then, the PI3K antagonist LY294002 was injected into the brain. Our results indicate that NMT can inhibit neuronal apoptosis, increase Bcl-2 expression, decrease caspase-3 and Bax expression, and downregulate the expression of PI3K, PDK1, AKT, and P-AKT. However, these changes can be reversed by LY294002. The results indicated that NMT may improve neuronal apoptosis in VD rats through PI3K/PDK1/AKT pathway, and the activation of PI3K/PDK1/AKT signaling pathway may be an important method for the treatment of cognitive decline.

This study explored the therapeutic effect of naomaitai on VD rats, providing a new idea for VD treatment. However, the specific components of naomaitai that play a protective role were not discussed, and the analysis of the specific components that have an effect could provide a more perfect strategy for VD treatment.

## 5. Conclusions

Naomaitai can ameliorate brain damage in rats with vascular dementia, inhibit neuronal apoptosis, and have anti-inflammatory and antioxidative stress effects, which may be regulated by the PI3K/PDK1/AKT signaling pathway.

## Data Availability

The data used to support the findings of this study are available from the corresponding author upon request.

## References

[1] O'Brien J. T., Thomas A. (2015). Vascular dementia. *The Lancet*.

[2] Benisty S. (2013). Current concepts in vascular dementia. *Geriatr Psychol Neuropsychiatr Vieil*.

[3] Maestre G. E., Mena L. J., Melgarejo J. D. (2018). Incidence of dementia in elderly Latin Americans: Results of the Maracaibo Aging Study. *Alzheimer's & Dementia*.

[4] Iadecola C. (2013). The pathobiology of vascular dementia. *Neuron*.

[5] Kalaria R. N. (2018). The pathology and pathophysiology of vascular dementia. *Neuropharmacology*.

[6] Lopez-Valdes H. E., Martinez-Coria H. (2016). The Role of Neuroinflammation in Age-Related Dementias. *Revista de Investigacion Clinica-Clinical and Translational Investigation*.

[7] Michaud M., Balardy L., Moulis G. (2013). Proinflammatory cytokines, aging, and age-related diseases. *Journal of the American Medical Directors Association*.

[8] Wang Z., Li Y.-M., Gong X.-J., Lin J., Zou J.-M. (2005). Effects of Naomaitai capsule on learning and memory and content of Ach in rat model produced by cerebral ischemia-reperfusion. *Zhongguo Zhongyao Zazhi*.

[9] Yang Q., Huang D. (2006). The Therapeutic Efficacy of Naomaitai Capsule on Lipid Level and Neural Function in Patients with Ischemic Stroke. *Chinese Journal of Integrative Medicine On Cardio-/Cerebrovascular Disease*.

[10] Chen J., Chen Z., Lin J. (2013). Cerebral paragonimiasis: A retrospective analysis of 89 cases. *Clinical Neurology and Neurosurgery*.

[11] Zhang C., Wang J. G., Zou W. X., Tian W. J. (2008). Clinical study on naomaltal capsule in treating lschemic cerebral infarction. *Guide of Chinese Medicine*.

[12] Chan Y., Wang M., Chen Y., Yang D., Lee M., Cheng F. (2003). Long-term administration of Polygonum multiflorum Thunb. reduces cerebral ischemia-induced infarct volume in gerbils. *American Journal of Chinese Medicine*.

[13] Cheon S. Y., Cho K. J., Lee J. E. (2013). Cerebroprotective effects of red ginseng extract pretreatment against ischemia-induced oxidative stress and apoptosis. *International Journal of Neuroscience*.

[14] Fei Y.-X., Wang S.-Q., Yang L.-J. (2017). Salvia miltiorrhiza Bunge (Danshen) extract attenuates permanent cerebral ischemia through inhibiting platelet activation in rats. *Journal of Ethnopharmacology*.

[15] Fu P. K., Pan T. L., Yang C. Y., Jeng K. C., Tang N. Y., Hsieh C. L. (2016). Carthamus tinctorius L. ameliorates brain injury followed by cerebral ischemia-reperfusion in rats by antioxidative and anti-inflammatory mechanisms. *Iranan Journal of Basic Medical Sciences*.

[16] Kim M., Bang J. H., Lee J. (2015). Salvia miltiorrhiza extract protects white matter and the hippocampus from damage induced by chronic cerebral hypoperfusion in rats. *BMC Complementary and Alternative Medicine*.

[17] Lee J. S., Choi H. S., Kang S. W. (2011). Therapeutic effect of Korean red ginseng on inflammatory cytokines in rats with focal cerebral ischemia/reperfusion injury. *American Journal of Chinese Medicine*.

[18] Lin G., Lin L., Liang H. (2010). Antioxidant action of a Chrysanthemum morifolium extract protects rat brain against ischemia and reperfusion injury. *Journal of Medicinal Food*.

[19] Zhang Y. Y., Li P. F., Li D. (2004). Effect of Ginkgo biloba leaf extract on electroencephalography of rat with cerebral ischemia and reperfusion. *Acta Pharmacologica Sinica*.

[20] Zou J.-M., Li Y.-M., Wang Z., Zhu H.-J., Zhou J. (2006). Effect of Naomaitai Capsule on learning and memory abilities and cerebral lipid-peroxidation in rat with vascular dementia. *Chinese Traditional and Herbal Drugs*.

[21] Pang X., Li T., Feng L., Zhao J., Zhang X., Liu J. (2014). Ellagic acid-induced thrombotic focal cerebral ischemic model in rats. *Journal of Pharmacological and Toxicological Methods*.

[22] Yoshida M. (1991). Neurocognitive Disorders Other Than Alzheimer Disease: Vascular Dementia. *Brain Nerve*.

[23] Lambert C., Zeestraten E., Williams O. (2018). Identifying preclinical vascular dementia in symptomatic small vessel disease using MRI. *NeuroImage: Clinical*.

[24] Valdés Hernández M. D. C., Booth T., Murray C. (2013). Brain white matter damage in aging and cognitive ability in youth and older age. *Neurobiology of Aging*.

[25] Shi S.-S., Yang W.-Z., Chen Y., Chen J.-P., Tu X.-K. (2014). Propofol reduces inflammatory reaction and ischemic brain damage in cerebral ischemia in rats. *Neurochemical Research*.

[26] Radak D., Katsiki N., Resanovic I. (2017). Apoptosis and acute brain ischemia in ischemic stroke. *Current Vascular Pharmacology*.

[27] Guo Y., Xu X., Li Q., Li Z., Du F. (2010). Anti-inflammation effects of picroside 2 in cerebral ischemic injury rats. *Behavioral and Brain Functions*.

[28] Solano-Gálvez S., Abadi-Chiriti J., Gutiérrez-Velez L. (2018). Apoptosis: Activation and Inhibition in Health and Disease. *Medical Sciences*.

[29] Adams J. M., Cory S. (2017). The BCL-2 arbiters of apoptosis and their growing role as cancer targets. *Cell Death & Differentiation*.

[30] Renault T. T., Dejean L. M., Manon S. (2017). A brewing understanding of the regulation of Bax function by Bcl-xL and Bcl-2. *Mechanisms of Ageing and Development*.

[31] Chen H., Ning X., Jiang Z. (2017). Caspases control antiviral innate immunity. *Cellular & Molecular Immunology*.

[32] Mirzayans R., Andrais B., Kumar P., Murray D. (2016). The Growing Complexity of Cancer Cell Response to DNA-Damaging Agents: Caspase 3 Mediates Cell Death or Survival?. *International Journal of Molecular Sciences*.

[33] Kim C., Park S. (2018). IGF-1 protects SH-SY5Y cells against MPP+-induced apoptosis via PI3K/PDK-1/Akt pathway. *Endocrine Connections*.

[34] Croze M. L., Soulage C. O. (2013). Potential role and therapeutic interests of myo-inositol in metabolic diseases. *Biochimie*.

[35] Ohtsubo K., Yamada T., Zhao L. (2014). Expression of Akt Kinase-interacting protein 1, a scaffold protein of the PI3K/PDK1/Akt pathway, in pancreatic cancer. *Pancreas*.

[36] Chang Y., Huang W., Sun Q. (2018). MicroRNA634 alters nerve apoptosis via the PI3K/Akt pathway in cerebral infarction. *International Journal of Molecular Medicine*.

[37] Zhu J., Wang J., Zhang X., Yu Y., Kang Z. (2018). Panax ginseng extract attenuates neuronal injury and cognitive deficits in rats with vascular dementia induced by chronic cerebral hypoperfusion. *Neural Regeneration Research*.

[38] Fan Y. Q., Cui J. C., Yang J., Wang P. (2018). Effect of Moxibustion on Delayed Memory and Expression of Hippocampal Nestin and Doublecortin Proteins in Dementia Rats. *Zhen Ci Yan Jiu*.

[39] Jia J., Wei C., Chen S. (2018). Efficacy and safety of the compound Chinese medicine SaiLuoTong in vascular dementia: A randomized clinical trial. *Alzheimers Dement (N Y)*.

[40] Luo X., Li A., Yang X. (2018). Paeoniflorin exerts neuroprotective effects by modulating the M1/M2 subset polarization of microglia/macrophages in the hippocampal CA1 region of vascular dementia rats via cannabinoid receptor 2. *Chinese Medicine*.

[41] Zou J. M., Pan Z. J., Wang S. L. (2003). Pharmacodynamic and Toxicologic Research on Nao Mai Kang Capsule. *China Journal of Traditional Chinese Medicine and Pharmacy*.

[42] Luo T. N., Yu X. L. (2013). Effect of Naomaitai Combined with Memantine on Vascular Dementia. *Acta Academiae Medicine Jiangxi*.

[43] Zhang H. N., Sun L., Tang Y. Y., Wang M. Y., Wu J. (2009). Effects of Naomaitai Capsule on cerebral blood flow and apoptosis of hippocampus neuron in rats with vascular dementia induced by chronic forebrain ischemia. *Journal of International Neurology and Neurosurgery*.

[44] Hasan T. F., Rabinstein A. A., Middlebrooks E. H. (2018). Diagnosis and Management of Acute Ischemic Stroke. *Mayo Clinic Proceedings*.

[45] Farkas E., Luiten P. G. M., Bari F. (2007). Permanent, bilateral common carotid artery occlusion in the rat: a model for chronic cerebral hypoperfusion-related neurodegenerative diseases. *Brain Research Reviews*.

[46] Braun M., Vaibhav K., Saad N. M. (2017). White matter damage after traumatic brain injury: A role for damage associated molecular patterns. *Biochimica et Biophysica Acta (BBA) - Molecular Basis of Disease*.

[47] Shi G., Liu C., Wang L., Guan L., Li S. (2012). Biomarkers of Oxidative Stress in Vascular Dementia Patients. *The Canadian Journal of Neurological Sciences*.

[48] Li Y., Zhang Z. (2015). Gastrodin improves cognitive dysfunction and decreases oxidative stress in vascular dementia rats induced by chronic ischemia. *International Journal of Clinical and Experimental Pathology*.

[49] Sinclair A. J., Bayer A. J., Johnston J. O., Warner C., Maxwell S. R. J. (1998). Altered plasma antioxidant status in subjects with Alzheimer's disease and vascular dementia. *International Journal of Geriatric Psychiatry*.

[50] Casado Á., López-Fernández M. E., Casado M. C., de la Torre R. (2008). Lipid peroxidation and antioxidant enzyme activities in vascular and Alzheimer dementias. *Neurochemical Research*.

[51] Shi G., Liu C., Li Q., Zhu H., Wang L. (2012). Influence of acupuncture on cognitive function and markers of oxidative DNA damage in patients with vascular dementia. *Journal of Traditional Chinese Medicine*.

[52] Wang M. M. (2018). Cadasil. *Handbook of Clinical Neurology*.

[53] Battisti C., Formichi P., Radi E., Malandrini A., Federico A. (2009). Evaluation of brain apoptosis in a CADASIL postmortem case. *Clinical Neuropathology*.

[54] Dieterle A. M., Böhler P., Keppeler H. (2014). PDK1 controls upstream PI3K expression and PIP3 generation. *Oncogene*.

[55] Lemmon M. A. (2007). Pleckstrin homology (PH) domains and phosphoinositides. *Biochemical Society Symposium*.

[56] Yadav S., Kalra N., Ganju L., Singh M. (2017). Activator protein-1 (AP-1): a bridge between life and death in lung epithelial (A549) cells under hypoxia. *Molecular and Cellular Biochemistry*.

[57] Yu Y., Zhang X., Li Z., Kong L., Huang Y. (2018). LncRNA HOTAIR suppresses TNF-*α* induced apoptosis of nucleus pulposus cells by regulating miR-34a/Bcl-2 axis. *Biomedicine & Pharmacotherapy*.

[58] Gagliardi P. A., Puliafito A., Primo L. (2018). PDK1: At the crossroad of cancer signaling pathways. *Seminars in Cancer Biology*.

[59] Poulsen A., Blanchard S., Soh C. K. (2012). Structure-based design of PDK1 inhibitors. *Bioorganic & Medicinal Chemistry Letters*.

